# Additive Manufacturing of Steel-Reinforced Concrete by Combination of Selective Paste Intrusion and Wire Arc Additive Manufacturing: Impact of Heat Generated by WAAM on Bond Behavior of the Reinforcement

**DOI:** 10.3390/ma18235455

**Published:** 2025-12-03

**Authors:** Alexander Straßer, Felix Riegger, Thomas Kränkel, Christoph Gehlen

**Affiliations:** 1 Centre for Building Materials (cbm), TUM School of Engineering and Design, Technical University of Munich, Lichtenbergstraße 2, 85748 Garching bei München, Germany; thomas.kraenkel@tum.de (T.K.); gehlen@tum.de (C.G.); 2 Institute for Machine Tools and Industrial Management (iwb), TUM School of Engineering and Design, Technical University of Munich, Boltzmannstraße 15, 85748 Garching bei München, Germany; felix.riegger@iwb.tum.de

**Keywords:** additive manufacturing, selective paste intrusion, wire arc additive manufacturing, bond strength, pull-out, directed energy deposition, temperature load, construction

## Abstract

Integrating Wire Arc Additive Manufacturing (WAAM) into the Selective Paste Intrusion (SPI) process represents a groundbreaking approach for fabricating reinforced concrete structures with complex geometries. This study investigates the bond strength between concrete and WAAM reinforcement under varying temperature conditions to understand the behavior of heated reinforcement bars within fresh concrete and its effect on the related bond strength. By conducting pull-out tests according to RILEM RC6, WAAM reinforcement bars were heated to predefined temperatures of 20 °C (ambient), 60 °C, 80 °C, and 200 °C for 18 min. The results show that while moderate thermal exposure (60 °C and 80 °C) led to a slight reduction in the maximum bond strength, a notable degradation occurred at 200 °C, indicated by a marked decrease in both maximum bond stress and early bond development. These findings provide initial insights into the thermal limitations of WAAM integration within SPI processes. The goal is to address the challenges associated with integrating WAAM into SPI, particularly the adverse effects of high temperatures generated during the welding process on the rheological properties of the cement paste, the penetration behavior of the paste in the particle bed, and ultimately, the mechanical properties of the hardened concrete. This technique allows for producing nearly free-formed reinforcements, thus complementing the advantage of SPI in producing free-formed structures of almost any geometry.

## 1. Introduction

The Selective Paste Intrusion (SPI) approach is an innovative additive manufacturing (AM) method designed specifically for producing concrete elements. According to DIN EN ISO/ASTM 52900:2022-03 [1], the Selective Paste Intrusion (SPI) process is classified within the Binder Jetting (BJT) category, as the bonding of the granular bed results from the selective deposition of a reactive binder. More specifically, SPI constitutes a multi-step binder-jetting process with chemically reactive bonding for cementitious materials (BJT-MSt/CRB/C). In this process, aggregates are first layered in a particle bed. Cement paste is then selectively applied to bind them. The surrounding unbound aggregates support the developing structure, enabling the production of geometrically complex elements without the need for formwork or additional support. After deposition, the structures are cured within the particle bed before excavation [2].

**Figure 1:**
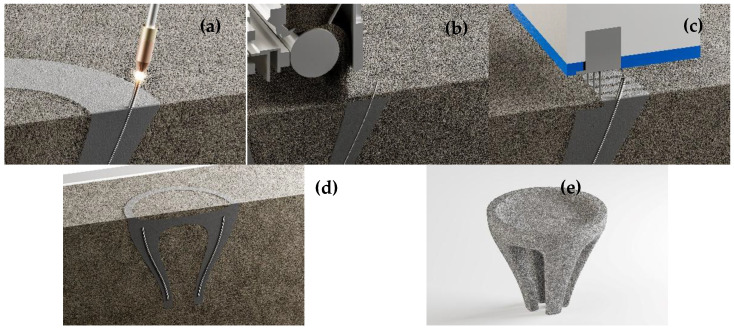
Combined SPI and WAAM process modified after [10]. (**a**) Printing a rebar, (**b**) spreading an aggregate layer, (**c**) cement paste application, (**d**) curing of the finished reinforced concrete element, and (**e**) excavated component.

A major challenge in additive manufacturing with concrete is the integration of reinforcement to produce elements that can resist not only compressive but also tensile and flexural stresses. Previous studies have shown that different reinforcement concepts are suitable for various AM processes in construction [3]. For particle-bed-based methods such as SPI, common strategies include placing reinforcement bars horizontally in the bed or inserting prestressing tendons or steel cables into predefined cavities. However, these approaches restrict geometric freedom. Combining SPI with an AM process capable of producing form-free, load-oriented steel reinforcement appears promising. Wire Arc Additive Manufacturing (WAAM) is an AM technique for producing metallic components and, according to DIN EN ISO/ASTM 52900:2022-03 [1], is classified as Directed Energy Deposition of metals using an electrical arc (DED-Arc/M). Due to its geometric flexibility and ability to process various metals, WAAM is used in aerospace (e.g., titanium, aluminum) [3], shipbuilding (e.g., steel, stainless steel) [4,5], power generation (e.g., nickel-based alloys) [6], and electrical engineering (e.g., copper alloys) [7]. Both low-alloy and highly ductile steels can be processed by WAAM [8,9], making it a suitable method for fabricating customized reinforcement within the SPI process without compromising geometric freedom. The combined SPI and WAAM process concept is shown in Figure 1.

The combined SPI-WAAM process begins with the layer-wise deposition of steel by WAAM, creating customized reinforcement directly inside the particle bed. A subsequent layer of aggregates is spread and leveled, followed by the selective intrusion of cement paste at predefined locations. This step locally binds the aggregates while embedding the reinforcement in the matrix. By repeating these steps, the concrete element is built layer by layer inside the bed through steel deposition, aggregate spreading, and paste intrusion. After fabrication, curing occurs within the surrounding aggregate bed, which acts as temporary formwork. Once curing is completed, the unbound aggregates are removed, and the finished reinforced element is excavated.

The released aggregates can be reused in subsequent builds. Initial investigations examined both the mechanical and bond behavior of WAAM reinforcements compared to conventional B500B rebars. Tensile tests on WAAM bars (Ø 12 mm, CMT-based WAAM process) showed yield strengths of 316 to 348 MPa, tensile strengths of 454 to 481 MPa, and uniform elongations of 14.4 to 17.5%, indicating sufficient ductility and strength for structural use, though slightly below B500B standards [11]. Despite lacking transverse ribs, WAAM bars achieved comparable bond performance in pull-out tests, which was attributed to their high surface roughness (R_q_ = approx. 100 µm), enabling effective mechanical interlock [11]. In contrast, other studies reported lower bond strengths (−14% to −26%) for additively manufactured steel rebars using gas metal arc welding compared to conventional reinforcement, despite the rough surface of the printed bars. Nevertheless, the results confirmed adequate bond performance for structural use, and the specimens exhibited enhanced ductility [12].

Integrating WAAM into the SPI process introduces specific challenges, particularly the high temperatures generated during the WAAM process. At the arc and melt pool, local temperatures exceed the melting point of steel (approximately 1425 to 1540 °C). These values occur above the particle bed and are not the governing condition for SPI integration. The relevant parameter is the thermal load transferred into the reinforcement segment embedded in the particle bed, where excessive heating may destabilize the aggregates and alter the rheology of the fresh cement paste. Previous research identified critical temperature thresholds. Cement paste above 60 °C exhibits a higher yield stress, which reduces its penetration performance [13]. Temperatures of 80 °C and above in the fresh state negatively affect the mechanical properties of the hardened concrete [14].

A study on passive cooling strategies investigated the effect of nozzle distance on temperature control within the WAAM rod. Larger nozzle distances reduce heat transfer to the reinforcement and the surrounding particle bed, as the gap between the welding point and the bed increases. A distance of 50 mm was identified as a practical compromise between mechanical strength, shape accuracy, and feasible cooling [10]. Depending on the cooling rate and distance to the welding point, the WAAM rod reaches temperatures between 100 °C and 450 °C, with approximately 200 °C measured at a 50 mm distance [10,15]. This temperature is considered representative for the SPI-WAAM interface, as it reflects the thermal load on the reinforcement section embedded in the particle bed at a practical process distance of 50 mm, while acknowledging that peak melt-pool temperatures at the welding point are significantly higher.

The process temperatures occurring during WAAM deposition for reinforcement fabrication within the SPI particle bed can exceed the temperature limits that are critical for the rheology and mechanical performance of cement paste [13,14]. This adversely affects bond strength. This can reduce bond strength. Previous investigations on bond behavior at elevated temperatures have primarily focused on fire exposure [16,17,18].

Although these studies provide insight into the mechanisms of bond degradation under heat, they describe a fundamentally different loading scenario. Fire tests typically involve uniform, long-term heating of entire concrete sections. In contrast, in WAAM, the thermal load is highly localized and inherent to the process, acting directly at the steel–concrete interface within the particle bed. To the authors’ knowledge, this localized, process-integrated heating and its effect on bond strength have not yet been systematically investigated. Nonetheless, fire-related studies remain a valuable reference for interpreting the observed bond response and serve as a comparative framework for the present work. The focus on process-integrated thermal loading thus represents the central contribution of this study. Based on these considerations, this study quantifies the influence of thermally induced conditions on the bond strength between WAAM reinforcement and concrete, taking into account previously identified critical temperature thresholds.

This study advances the previous research presented in [14,15] by experimentally quantifying the direct effect of localized, process-integrated thermal exposure from WAAM on the bond behavior between steel reinforcement and concrete. In contrast to [14], which addressed external thermal loading and its impact on hardened-state strength, and [15], which investigated WAAM temperature profiles and passive cooling strategies, the present work focuses on the bond mechanisms under realistic, embedded thermal conditions representative of combined SPI and WAAM production.

## 2. Materials and Methods

### 2.1. Methods and Sample Preparation

Since standardized pull-out samples according to RILEM RC6 [19] cannot currently be produced using SPI or combined SPI and WAAM processes due to current process limitations, the specimens were conventionally cast using formwork. The mix design and volumetric ratio of paste and aggregates were adjusted to closely match those in SPI-fabricated elements. For this, the same cement paste formulation was employed. The same cement paste formulation was used, and the aggregate-to-paste ratio was matched to that in the SPI particle bed by recalculating the theoretical volume ratio between aggregate and paste. In SPI, this ratio is achieved by pouring a loose aggregate bed and selectively infiltrating the voids with cement paste. A direct pore-structure comparison was beyond the scope of this study, but previous SPI investigations [13,14] indicated similar matrix density and particle packing. Differences in compaction, porosity distribution, and the interfacial transition zone may inherently arise between conventionally cast and SPI-formed structures. Previous studies have also shown that SPI-fabricated specimens exhibit isotropic material properties and comparable elastic modulus and strength to conventionally cast specimens produced with the same mix design [2]. Despite the current constraints in specimen preparation, the results are considered representative and relevant for both SPI and combined SPI-WAAM systems. SPI-fabricated pull-out specimens are planned for future validation.

**Figure 2:**
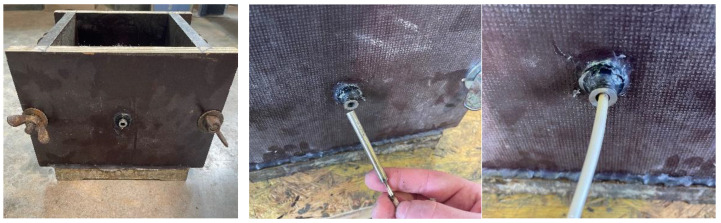
Inserting the heating cartridge into the drilled core hole of the WAAM rebar.

A preliminary test series determined how a defined temperature load could be transferred from the reinforcement bar to the surrounding concrete [20]. The WAAM rebars were longitudinally drilled on a lathe to create a core hole with a diameter of 6.1 mm and a depth of 90 mm. A cartridge heater was inserted and controlled by a temperature controller (Shinko GCS, Nagano, Japan) operated in on/off mode, as shown in Figure 2.

In the combined SPI-WAAM system, the surface temperature of the steel bar is decisive, as it directly affects the surrounding concrete. Although heating in this experiment was applied internally via a cartridge inserted into the bar, the high thermal conductivity of steel ensured that nearly identical temperatures were achieved at the surface of the bar.

Measurements confirmed that the temperature difference between the bar core and surface was negligible within the ranges investigated (60 °C, 80 °C, and 200 °C). Direct surface instrumentation was avoided, since casting fresh concrete at approximately 20 °C around surface sensors would have distorted the measurements.

The core-hole depth was selected to ensure that the heating cartridge, despite a 20 mm thick formwork, could be placed deep enough to cover the entire effective bond length with the heated zone. For specimen fabrication, the WAAM reinforcement bar was centrally placed in a cubic formwork with an edge length of 200 mm, and the embedded bar length in the bond was 60 mm (5 × d_s_ with d_s_ = 12 mm being the diameter of the rebar), as per RILEM RC6 [19].

**Figure 3:**
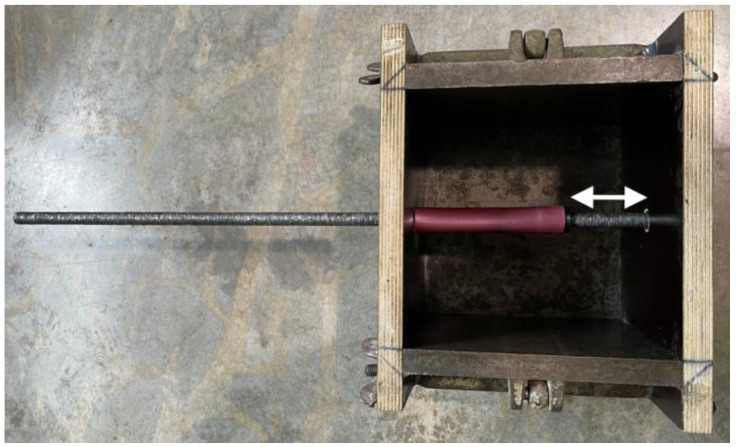
Placement of the WAAM rebar in the formwork: The non-embedded steel area is covered with a hose (purple). The embedded bar length of 60 mm is marked with a white arrow.

The remaining embedded length not intended to participate in the bond (140 mm) was isolated with a flexible hose (inner diameter d_s_ + 2 mm), as shown in Figure 3.

All specimens were compacted using a concrete vibrator (Wacker Irsen 18 mm 200 Hz, Munich, Germany) for 30 s. Immediately after compaction, the heating cartridge was inserted into the drilled WAAM rebar, and thermal loading commenced without delay. The bonded steel was then heated to a defined temperature. Due to the high power density of the cartridge heater, the target temperatures were reached within seconds after activation. The selected setpoints (60 °C, 80 °C, and 200 °C) were based on critical temperature thresholds reported in previous studies [13,14,15].

Once the setpoint was reached, it was held for 18 min to approximate the thermal exposure that a 60 mm bond segment experiences during WAAM production under the present parameters. While the cyclic heating/cooling inherent to WAAM could not be reproduced with this setup, continuous heating over the same time span provides the best available approximation of the process-integrated heat input. Specimens were demolded after 2 days and stored at 20 °C/65% relative humidity until testing after 28 days.

For each temperature setpoint, three pull-out specimens were produced. In parallel, six prisms according to DIN EN 196-1:2016-11 [21] with dimensions of 40 × 40 × 160 mm^3^ were cast from multiple concrete batches and tested at 28 days for tensile and compressive strength to verify batch comparability.

**Figure 4:**
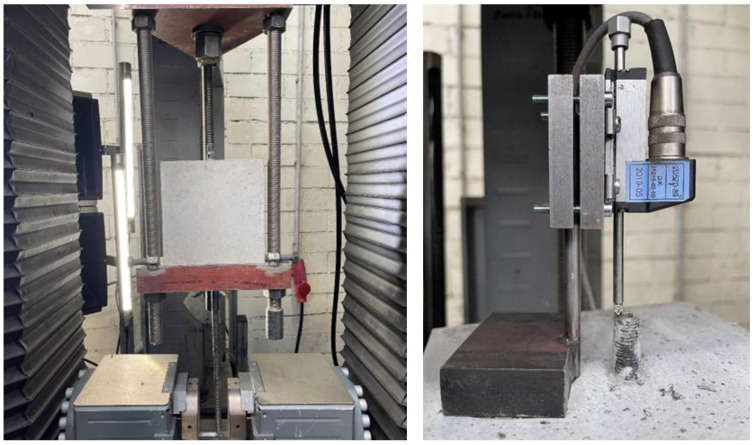
Test setup in the universal testing machine, showing the concrete specimen with the WAAM reinforcement bar extending vertically downward through the loading fixture (left). The displacement transducer mounted at the free (non-loaded) end of the reinforcement to record the slip during loading is shown as a detailed view (right).

Pull-out tests were conducted according to RILEM RC6 [19]. Specimens were positioned on the support platen of a universal testing machine (Zwick Z600, Ulm, Germany) with the WAAM bar protruding vertically downward. Displacement was recorded at the unloaded bar end using a displacement transducer (FWA025TR, Ahlborn, Holzkirchen, Germany) mounted via a rigid bracket epoxied to the concrete, as shown in Figure 4.

Load was applied to the bar end at a rate of 72 N/s (0.5 × d_s_^2^), in accordance with RILEM RC6 [19]. The bond behavior was assessed from the bond-stress–displacement response at predefined displacements of 0.001 mm, 0.01 mm, and 0.1 mm, as well as at the peak bond stress. The temperature groups were compared accordingly. Prisms were cast for each batch to monitor potential variations in material properties from one batch to the next.

To complement the mechanical bond tests, additional microstructural and compositional analyses were carried out. X-ray diffraction (XRD) was performed to quantify the crystalline and amorphous phase composition of the interfacial transition zone (ITZ) and to identify temperature-dependent changes in hydration and carbonation using a Bruker D8 ADVANCE 145 (Billerica, MA, USA) with a θ-θ configuration and CuKα radiation (λ = 1.54 Å, 40 kV).

Thermogravimetric analysis (TGA) was carried out on a Netzsch STA 449 F3 Jupiter (Selb, Germany) using a heating rate of 10 K/min under a nitrogen flow of 20 mL/min. Approximately 25 ± 2 mg of sample was weighed into an 85 µL corundum crucible. Mercury intrusion porosimetry (MIP) was performed to determine the pore size distribution and total porosity of the matrix, combining a low-pressure measurement (up to 100 kPa) using a Microtrac BELPORE LP (Software: PoreInspect) and a high-pressure measurement (100 kPa to 414 MPa) using a Microtrac BELPORE HP.

### 2.2. Materials

The mix design of the concrete used for the pull-out specimens is summarized in Table 1. The concrete used for the pull-out specimens was produced with ordinary Portland cement (strength class 42.5 N, density approximately 3.10 kg/m^3^) and quartz sand with a particle size ranging from 1.0 to 2.2 mm (density approximately 2.65 kg/m^3^). The water-to-cement ratio (w/c) was 0.40. The volumetric composition consisted of 42 vol.% cement paste and 58 vol.% aggregates. A polycarboxylate-ether-based superplasticizer was used to adjust the flow properties of the cement paste, targeting a mini-slump flow of 400 to 410 mm.

**Figure 5:**
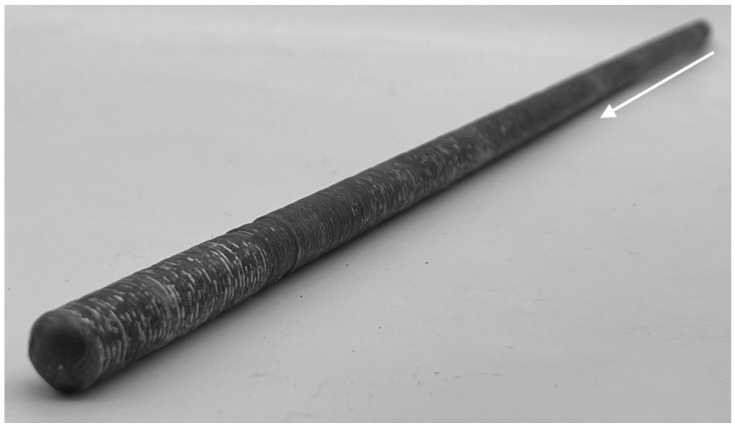
Rebar with a nominal diameter of 12 mm manufactured using the WAAM process. The building direction (z-direction) is marked with an arrow.

Rebars with a nominal diameter of 12 mm were manufactured using WAAM with the Cold Metal Transfer (CMT) cycle step process, as shown in Figure 5. The building direction (z-direction) is marked with an arrow.

The applied process parameters as well as the resulting mechanical performance are listed in Table 2. This data originates from a previous study [11], produced with the same machine, parameters, and feedstock material; hence, they are considered directly representative for the present work.

## 3. Results

**Figure 6:**
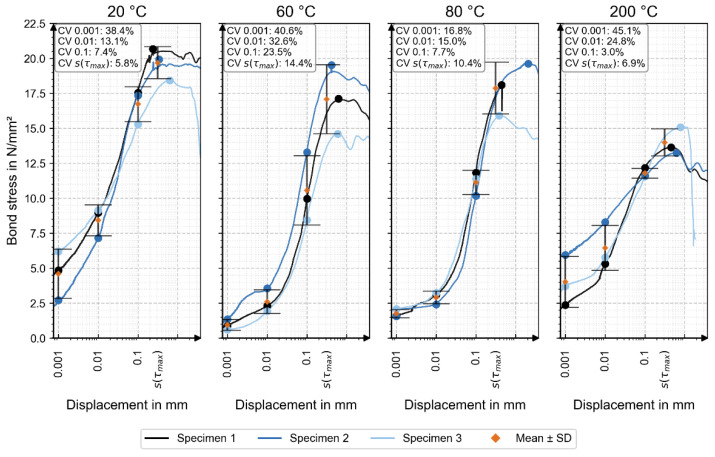
Correlation between bond stress and displacement for the three specimens of each temperature series, including the mean value with standard deviation. The partial overlap of markers reflects the close agreement of the individual measurements.

Detailed numerical results are provided as supplementary research data [22]. Figure 6 shows the individual bond-stress–displacement curves for all specimens. Table 3 shows the mean values of the bond stress depending on displacement and temperature load. The corresponding mean values (orange) and standard deviations are also included, with coefficients of variation (CV) indicated for each displacement level.

The reference at 20 °C (without thermal loading) exhibited the highest bond stress at every measured displacement value, as well as at the maximum bond stress.

This suggests that thermal loading of the reinforcement bar has a negative impact, although no consistent trend was observed. For displacement values of 0.001 mm and 0.01 mm, the thermally loaded samples at 60 °C, 80 °C, and 200 °C showed increasing bond strength with rising temperature. At a displacement of 0.1 mm, the bond stress values for the thermally loaded samples ranged between 10.56 N/mm^2^ and 11.78 N/mm^2^, indicating comparable bond performance [22]. The maximum bond stress at 60 °C and 80 °C slightly decreased compared to the reference, but remained relatively stable, while a notable reduction in both maximum bond stress and peak load was observed at 200 °C.

All specimens failed due to bond loss, as identified by bar pull-out without steel yielding or concrete splitting. The geometry complied with RILEM RC6 [19], providing sufficient cover (cube edge length 200 mm, ≥10 × d_s_ with d_s_ = 12 mm). Thus, splitting was not expected or observed. The variation in bond stress with temperature indicates a displacement-dependent mechanism and a complex interaction between thermal effects and local failure modes.

The load–displacement curves followed a consistent sequence: an initial adhesion-controlled stage, followed by shear bond as the main load-transfer mechanism, and a final frictional stage with progressive decay until pull-out failure. This pattern matches the classical bond behavior of ribbed reinforcement in pull-out tests, confirming that shear bond governed load transfer. Only specimen 80-1 deviated, showing minimal residual friction and a sharp post-peak drop.

The average tensile strength of the concrete prisms was 8.1 MPa, and the compressive strength was 72.4 MPa, tested on 40 × 40 × 160 mm^3^ prisms according to DIN EN 196-1:2016-11 [21]. With a standard deviation of 0.6 N/mm^2^ in tensile strength and 3.3 N/mm^2^ in compressive strength, the mechanical properties of the concrete mixes were within a comparable range. Therefore, we assume that there were no significant differences within the concrete mixtures for the pull-out tests, and consequently, no significant impact on the pull-out results.

Previous studies on conventional reinforcement exposed to high temperatures, mainly in fire tests, report a general decrease in bond strength with increasing temperature [16,17,18,23,24]. Some studies, however, have observed a temporary increase up to 450 °C, followed by a decline at higher levels [18,24]. These behaviors are attributed to altered deformation and increased slip at lower loads, collectively indicating thermal degradation of bond stiffness and capacity. In contrast to such fire-exposure conditions, the present study reproduces localized, process-integrated heating at the steel–concrete interface during fresh-state deposition, a fundamentally different scenario.

To interpret the observed mechanical trends, complementary microstructural analyses (XRD, TGA, MIP) were conducted. Quartz originating from the sand fraction was computationally removed from the spectra to better assess binder phases.

**Figure 7:**
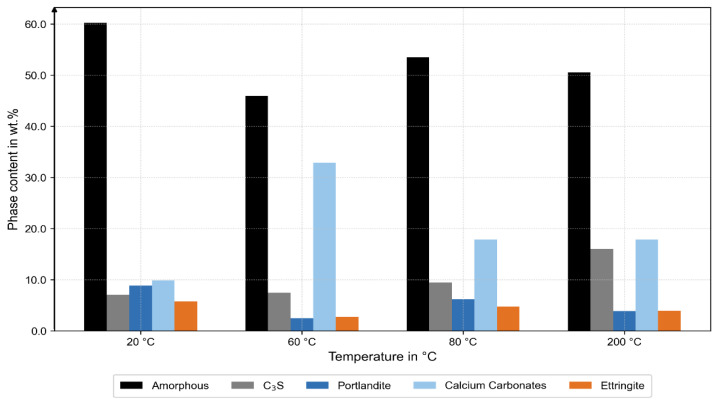
XRD analysis. Increased C_3_S at 200 °C (grey), decreased amorphous content at 60 °C (black), and higher carbonate content at 60 °C (light blue).

After correction, the specimens at 20 °C, 60 °C, and 80 °C exhibited generally comparable amorphous contents; at 60 °C, however, the amorphous fraction was slightly lower and accompanied by a minimum in portlandite and higher carbonate contents (calcite and vaterite), as shown in Figure 7. This indicates enhanced carbonation and locally hindered hydration, likely caused by early water expansion or micro-cracking in the ITZ during thermal exposure and subsequent storage after the bond tests.

At 200 °C, a significantly higher fraction of unhydrated C_3_S was observed, indicating limited hydration and partial dehydration [25]. Ettringite was detected in all specimens, even in that at 200 °C, although this phase is thermally unstable above approximately 60 °C. This suggests secondary formation upon cooling, driven by rehydration from residual moisture.

**Figure 8:**
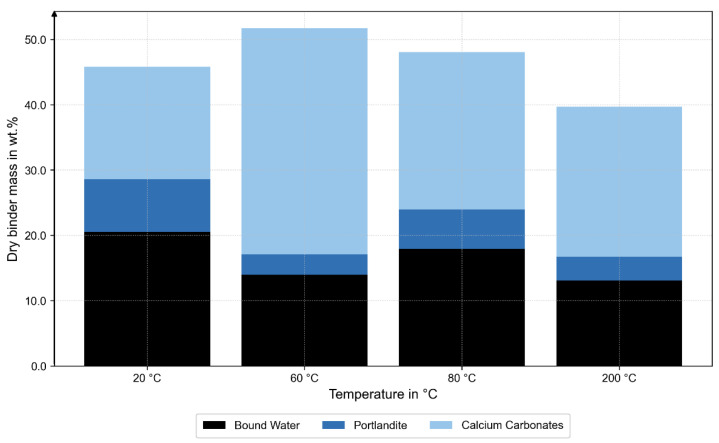
TGA confirms the XRD analysis in carbonate content at 60 °C and dehydrated C-S-H phases at 200 °C.

The TGA results confirm these trends: at 60 °C, the smallest loss of portlandite was observed, while the 200 °C specimen exhibited a pronounced mass loss in the range of 50 to 350 °C, indicating dehydration of the C-S-H phases (see Figure 8).

**Figure 9:**
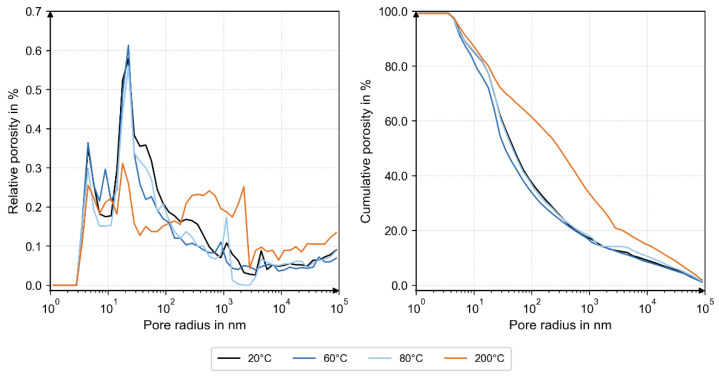
MIP analysis. Left: relative porosity; right: cumulative porosity. Nearly similar pore size distribution at 20 °C, 60 °C, and 80 °C. Shifted pore size distribution to larger pore radii (left), and increased total porosity (right) at 200 °C.

MIP analysis revealed nearly identical pore-size distributions and total porosity up to 80 °C. At 200 °C, the distribution shifted toward larger pore radii, demonstrating thermally induced micro-cracking and matrix loosening (Figure 9).

These observations from the MIP, XRD, and TGA analyses are consistent with the mechanical results. The maximum bond stress (τ_max_) remained comparable at 20 °C, 60 °C, and 80 °C, despite the reduced amorphous and portlandite fractions at 60 °C, indicating only minor changes in the hydration state. Up to 80 °C, τ_max_ is primarily governed by shear transfer in the concrete ribs and bulk porosity, both of which remain effectively unchanged. At 200 °C, τ_max_ decreases to 13.99 N/mm^2^, consistent with partial dehydration and matrix coarsening observed microstructurally. Moderate thermal exposure up to 80 °C had little influence on peak bond strength, whereas heating to 200 °C caused the decomposition of hydration products, coarsening of pore structures, and a reduction in load-bearing capacity.

At small displacement levels (<0.01 mm), the 60 °C and 80 °C specimens showed lower bond stresses than the reference, while the 200 °C specimens reached comparable values. This trend may be linked to heat-induced water evaporation at the steel–concrete interface, which locally reduces the water-to-cement ratio, increases matrix stiffness, and promotes more brittle behavior. Bond stresses rose from 0.94 to 4.01 N/mm^2^ (τ_0.001_) and from 2.60 to 6.45 N/mm^2^ (τ_0.01_) between 60 °C and 200 °C, indicating increased resistance to micro-displacement but reduced ductility. At higher displacements (0.1 mm), bond stresses remained nearly constant (10.56 to 11.78 N/mm^2^), while τ_max_ declined at 200 °C, reflecting the earlier onset of damage and limited energy dissipation.

The microstructural results therefore suggest a temperature-dependent stiffening of the bond interface at early loading stages, governed primarily by chemical and microstructural effects in the interfacial transition zone (ITZ). With increasing displacement, mechanical interlocking and matrix porosity become dominant, resulting in overall bond weakening at elevated temperatures. This interpretation is consistent with the observed trends in MIP, XRD, and TGA but remains hypothetical, as the current methods do not yet allow a definitive causal link between ITZ composition and mechanical response. Further high-resolution characterization of the ITZ would be required to verify these mechanisms.

## 4. Conclusions and Outlook

In summary, the results indicate that thermal loading of the reinforcement bar affects the bond behavior in a displacement-dependent manner. While the reference samples at 20 °C achieved the highest bond stress across all displacement values and the maximum bond strength, thermally exposed samples exhibited a general reduction in performance. Nevertheless, moderate thermal exposure (60 °C and 80 °C) still maintained a comparable bond strength at higher displacements (0.1 mm), suggesting that the adverse effects may be more pronounced in the early stages of load transfer. The observed trends support the hypothesis that thermal effects alter the micro-structure in the bond zone, potentially leading to increased brittleness and local strength at low displacements, followed by premature degradation upon further loading. This is particularly important because, in practical applications of reinforced concrete structures, such small displacements are typically decisive for the onset of failure.

The complementary XRD, TGA, and MIP analyses provide microstructural evidence for these mechanisms. XRD revealed temperature-dependent changes in phase composition: at 200 °C, a higher fraction of unhydrated C_3_S indicated limited hydration and partial dehydration of the binder matrix, while at 60 °C, a reduced amorphous C-S-H fraction accompanied by a minimum in portlandite and increased carbonate contents (calcite, vaterite) suggested early carbonation. The TGA results confirmed these observations, showing higher carbonate-related mass loss at 60 °C and dehydration of C-S-H phases at 200 °C. Together, these findings indicate a progressive transformation from a hydrated to a partially dehydrated and carbonated state with increasing temperature. The MIP data complement this interpretation: pore size distributions at 20 °C, 60 °C, and 80 °C were nearly identical, while the 200 °C sample exhibited larger pore radii and higher total porosity.

While moderate temperatures (60 °C and 80 °C) were expected to have a limited impact on bond strength, the behavior at 200 °C was unexpected. It showed an increase in early bond stiffness accompanied by a significant reduction in maximum bond capacity. Contrary to the anticipated trend of steadily decreasing bond strength with rising temperature, this result points to a complex thermal effect on the ITZ. Thermal exposure may locally enhance micro-level stiffness while simultaneously promoting premature bond failure due to changes in the ITZ microstructure. These findings emphasize the crucial importance of effective thermal management during WAAM integration.

This study primarily assessed global bond behavior under controlled thermal exposure. Future work will focus on three aspects: (i) conducting pull-out tests on SPI-fabricated specimens to confirm the transferability of these findings to process-integrated conditions; (ii) implementing real-time thermal monitoring during WAAM integration to directly correlate temperature histories with bond response; and (iii) verifying the hypothesized mechanisms by microstructural sectioning and high-resolution microscopy. Advanced ITZ characterization, including phase mapping and nanoscale imaging, will be critical to substantiate the observed transformations and to establish a mechanistic understanding of thermally induced bond degradation.

## Figures and Tables

**Table 1:** Mix design of concrete with w/c 0.4 used for pull-out specimens.

Material/Parameter	Unit	Amount
Ordinary Portland Cement	kg/m^3^	580
Water	kg/m^3^	232
Quartz sand (1.0 to 2.2 mm)	kg/m^3^	1537
Superplasticizer (Polycarboxylate-ether)	% bwoc	0.8

**Table 2:** Material, parameters, and specifications of the process and produced WAAM rebars [11].

Material/Parameter	Unit	Value/Specification
Welding process	-	CMT cycle step (Fronius TPS400i)
Substrate	-	S235JR
Electrode wire (to produce WAAM rebar)	-	G4Si1
Welding speed	m/min	0.25
Wire feed speed	m/min	3.2
Cooling time per layer	s	96
Upper yield stress	N/mm^2^	390
Tensile strength	N/mm^2^	481
Uniform elongation	%	16.0
Young’s modulus	N/mm^2^	187,000

**Table 3:** Mean values of bond stress depending on displacement and temperature load.

	τ_0.001_ in N/mm^2^	τ_0.01_ in N/mm^2^	τ_0.1_ in N/mm^2^	τ_max_ in N/mm^2^
20 °C	4.59	8.42	16.72	19.67
60 °C	0.94	2.60	10.56	17.08
80 °C	1.84	3.15	11.60	17.00
200 °C	4.01	6.45	11.78	13.99

## Data Availability

The data supporting the results of this study are available on Zenodo at https://doi.org/10.5281/zenodo.16562375 [22]. The data are currently under embargo until the publication of the associated article. Upon publication, the data will be publicly accessible under the Creative Commons Attribution 4.0 International (CC-BY 4.0) license.
